# Dietary supplementation of low levels of unprocessed *Mucuna pruriens utilis* seed meal induces mild antinutritional entero-physio-metabolic perturbations without compromising performance and meat quality in finisher broilers

**DOI:** 10.1007/s11250-023-03760-8

**Published:** 2023-09-28

**Authors:** Pretty Ntombizethu Zungu, Doctor Mziwenkosi Nhlanhla Mthiyane, Sithandiwe Eunice Mazibuko-Mbeje, Mercy Chisara Ogwuegbu

**Affiliations:** 1 Department of Animal Science, School of Agricultural Sciences, Faculty of Natural and Agricultural Sciences, North-West University (Mahikeng Campus), Private Bag X 2046, Mmabatho, 2735 South Africa; 2 Food Security and Safety Focus Area, Faculty of Natural and Agricultural Sciences, North-West University (Mahikeng Campus), Mmabatho, 2735 South Africa; 3 Department of Biochemistry, North-West University, Mmabatho, 2745 South Africa; 4 Department of Life and Environmental Sciences, Polytechnic University of Marche, 60131 Ancona, Italy; 5 Department of Animal Science, Faculty of Agriculture, University of Nigeria, Nsukka, Enugu State 410001 Nigeria

**Keywords:** Legume, Alternative protein source, Phytogenic feed additive, Meat production and quality

## Abstract

The rapidly growing world human population accentuates the need for improved production especially of protein-rich food. Broiler meat production offers opportunity to ensure security of this food. However, the production of modern broilers is not only limited by high feed costs due to dietary use of expensive energy and protein sources but also their meat possesses undesirable quality attributes. This study thus examined the effect of dietary *Mucuna pruriens utilis* seed meal (MSM) on growth performance, blood profile, carcass traits, and meat quality in finisher broiler chickens. In a completely randomised design (CRD), 320 21-day-old chicks were randomly allocated to 32 pens in which they were allotted 4 dietary treatments with 0, 2.5, 5, and 10% MSM, each with 8 replicate pens of 10 birds, for 28 days. Growth performance, carcass characteristics, internal organs, haemato-biochemistry, and meat quality were measured. Results showed that dietary MSM did not affect (*P* > 0.05) broiler performance, weights, and lengths of carcass cuts and internal organs, haematology, and meat quality. The only exception was MSM-induced increase in duodenal weight (linear, *P* < 0.05) and serum phosphorus (quadratic, *P* = 0.05) in contrast to a decrease in procalcitonin (quadratic, *P* < 0.01) and serum levels of total protein (linear, *P* < 0.05; and quadratic, *P* < 0.01), albumin (quadratic, *P* < 0.05), and bilirubin (quadratic, *P* = 0.001). Therefore, MSM could be supplemented up to 10% without compromising performance, carcass traits, internal organs, haemato-biochemistry, and meat quality in finisher broiler diets.

## Introduction

The world human population continues to grow rapidly and is envisaged to reach between 9.4 and 10.1 billion by 2050 (United Nations, 2019). This concomitantly increases the demand for food and thus accentuates the need for strategies to improve production especially of protein-rich food to eradicate both food insecurity and malnutrition particularly in low- and middle-income countries of Africa (Ayodele et al., 2021; OECD/FAO, 2021). Considering its fastest global growth among all agricultural subsectors (Research and Market, 2019), its perception as being relatively nutritionally valuable with a preponderance of health-desirable unsaturated fatty acids (Barroeta, 2007; Cavani et al., 2009), as well as its affordability to most consumers (Valceschini, 2006), broiler meat is the most strategic type of protein-rich food for which development interventions are required to enhance its capacity and efficiency of production.

Notwithstanding, the production of broiler chickens is limited by high feed costs. These represent about 65–75% of total variable costs (Panda et al., 2014), of which about 95% are ascribable to energy and protein (plant and animal derived) sources (Mallick et al., 2020). To decrease these costs, replacement of expensive energy and protein ingredients with cheaper and locally abundant alternative feedstuffs, among other options, has been the most prudent strategy to sustain broiler production (El-Deek et al., 2020). Particularly, the focus of replacement has been directed at maize and soya bean meal (SBM), the major dietary plant-derived protein sources commonly used in broiler diets (Khalil et al., 2023). Aside exorbitant feed costs, modern fast-growing broiler strains produce meat with undesirable quality attributes imputable to intensive genetic selection and breeding programmes over the past decades. They deposit excessive abdominal fat (Tumova et al., 2021), and produce meat with high fat content and low pH (Kocer et al., 2018). The pH of meat is an important quality characteristic, the decline of which is associated with paler and softer meat with higher drip loss. Drip loss is visually unattractive to consumers and can result to excessive cooking losses and cooked meat dryness (Marchewka et al., 2023). Therefore, there is a need for strategies to improve not only production but also the quality of broiler meat to ultimately improve its appeal among consumers. One of the alternative feedstuffs with nutritional attributes that qualify it for partial replacement of maize and soya bean meal in broiler diets, as well as bioactive compounds with great potential to enhance desirable quality properties of the modern birds’ meat, is MSM.

The seeds of *Mucuna pruriens* (L.) DC var. *utilis* (Wall. ex Wight) Baker ex Burck (a.k.a velvet bean), a tropical/sub-tropical legume, are rich in proteins (25–30 g/100g) and starch (39–41 g/100g) (Ezeagu et al., 2003) as well as essential amino acids lysine, methionine, and others (Kouakou et al., 2022). They are also endowed with a plethora of bioactive phytochemical compounds primary among which is a high content (≤ 9%) of 3,4-dihydroxy-L-phenylalanine (L-DOPA) (Pulikkalpura et al., 2015; Daffodil et al., 2016; Konishi et al., 2022). L-DOPA is a precursor for dopamine and other catecholamines (epinephrine and norepinephrine) (Molinoff and Axelrod, 2022; Weiner, 1979) that physiologically behave as β-agonists due to their enhancement of muscle accretion (Navegantes et al., 2000). Hence, consumption of MSM induces anabolic effects and increases muscle mass mediated through its ability to increase growth hormone (Alleman et al., 2011), testosterone (Suresh and Prakash, 2012), and dopamine (Lieu et al., 2012). Also, consumption of MSM has been demonstrated in poultry and rodent studies to induce hypoglycemic, lipid, and low-density lipoprotein cholesterol lowering effects (Jayaweera et al., 2007; Dharmarajan et al., 2012; Ngatchic et al., 2016). Further, MSM incorporation into beef diets has been shown to decrease meat cooking losses (Yantika et al., 2016).

Notwithstanding, due to its endowment with L-DOPA, dietary intake of MSM induces weight loss and decreases feed intake and feed conversion efficiency in non-ruminants including broilers (Del Carmen et al., 2002; Flores et al., 2002) especially when included at high levels (≥ 15%) (Vadivel and Pugalenthi, 2010a). For this reason, it may be prudent to include MSM at low levels (≤ 10%) in broiler diets as it has recently been recommended (Ayodele et al., 2021) as a strategy to impart its anabolic effects on broiler meat as it was shown in mice (Imbs and Schwartz, 2011) whilst minimising the likelihood of its toxicity. Therefore, the objective of this study was to investigate the growth performance, carcass characteristics, internal organs, haemato-biochemistry, and meat quality in finisher broilers fed low-graded levels of unprocessed MSM as a partial replacement for maize and SBM as well as a phytogenic feed additive.

## Materials and methods

### Study site and ethical consideration

The protocols used in this study were approved by the North-West University (NWU) Animal Production Sciences Research Ethics Committee (NWU-00814-31-AS). The study was conducted using broiler facilities at the NWU Experimental (Molelwane) farm (coordinates: 25° 40.459′ S, 26° 10.563′ E) situated in the Mahikeng Local Municipality, North-West Province, South Africa.

### Sourcing of *M. pruriens utilis* seeds and other ingredients, diet formulation, and chemical analysis

*Mucuna* seeds were purchased from TWK (Piet Retief, RSA), whilst all other dietary ingredients were procured from Optifeeds (PTY) LTD., Lichtenburg, RSA. The experimental diets were formulated at SimpleGrow Agric Services (Pty) Ltd. (Irene, Gauteng, South Africa) to produce 5 iso-caloric and iso-nitrogenous (18% CP) diets with maize, SBM, and full fat soya bean being partially substituted with incremental inclusion levels of MSM (0, 2.5, 5, and 10%) to meet nutritional requirements of finisher broilers (d22–49) (Table 1) (NRC, 2001). Then, 100 g samples of MSM and experimental diets were milled (1 mm screen) and analysed for DM (930.15), CP (954.01), EE (920.39), ash (942.05), and CF (AOAC, 2005). Their DM was determined by oven-drying (105 °C) 1 g of sample in pre-weighed crucibles for 12 h followed by cooling in a desiccator and weighing. It was then calculated as a difference between initial sample weight and moisture weight. CP was determined following the Kjeldahl method and multiplying nitrogen values by 6.25. EE was determined by ether extraction of crude fat using an automated Soxhlet Fat analyser ANKOMXT15 extractor following the operator’s manual (ANKOM Technology, Macedon, NY, USA). Ash was determined by calcinating a dry 1 g sample in the muffle furnace (550 °C) (Nabertherm GmbH, Germany) for 6 h. CF was analysed using the ANKOM2000 Fibre Analyzer with 0.255 N crude fibre acid solution and 0.313 N crude fibre base solution. Also, NDF, ADF, and ADL were analysed following protocols of Van Soest et al. (1991). Both NDF and ADF were analysed using the automated ANKOMDELTA fibre analyser (ANKOM Technology, Macedon, NY, USA), whilst ADL was determined by immersing ADF residue bags for 3 h in 72% (wt/wt) sulphuric acid.

**Table 1:** Ingredient, chemical, and amino acid composition (%) of experimental finisher diets

Ingredient	Dietary MSM inclusion level				
	0%	2.5%	5%	10%	MSM
Yellow maize	64.3	63.5	62.8	61.2	-
Soya bean oil cake (47%)	18.0	17.6	17.3	16.7	-
Full fat soya bean	14.0	12.6	11.2	8.3	-
Mucuna seed meal	0.00	2.50	5.00	10.0	-
Mono-calcium phosphate	1.35	1.36	1.37	1.40	-
Limestone	1.28	1.24	1.20	1.13	-
Broiler finisher premix	0.25	0.25	0.25	0.25	-
Sodium bicarbonate	0.18	0.19	0.20	0.21	-
DL-Methionine (98%)	0.20	0.21	0.22	0.25	-
Lysine (78%)	0.10	0.14	0.17	0.23	-
Threonine (98%)	0.03	0.04	0.05	0.08	-
Salt	0.31	0.30	0.30	0.28	-
*Chemical composition*					
Dry matter	93.4	84.5	88.7	88.6	90.6
Crude protein	18.0	18.0	18.0	18.0	31.4
Ether extract	7.60	6.60	7.20	7.20	7.4
Ash	6.40	6.20	6.10	6.40	4.7
Crude fibre	3.40	3.50	3.60	3.70	-
Neutral detergent fibre	38.2	29.9	39.1	35.8	33.6
Acid detergent fibre	22.1	28.4	29.9	25.7	11.1
Acid detergent lignin	6.60	9.30	10.5	14.0	8.16
*Amino acid composition*					
Lysine	10.3	10.3	10.2	10.2	-
Methionine	4.70	4.80	4.80	4.90	-
Cysteine	3.00	2.90	2.90	2.70	-
Methionine + cysteine	7.80	7.70	7.70	7.60	-
Threonine	7.10	7.10	7.00	7.00	-
Tryptophan	2.00	2.00	1.90	1.80	-
Isoleucine	7.60	7.30	7.00	6.40	-
Arginine	12.1	11.6	11.2	10.4	-
Valine	8.60	8.30	8.10	7.70	-
Glycine + serine	19.7	19.6	19.4	19.1	-

*MSM*, mucuna seed meal

### Study design and broiler management

Day-old Cobb 500 male broiler chicks purchased from Eagle Pride Hatchery were initially ad libitum fed a commercial starter diet from d1 to 21, after which they were introduced to experimental finisher diets from d22 to 49. They were supplied with StressPack with electrolytes and vitamins for 48 h. In a CRD, 320 21-day-old chicks were randomly allocated to 32 pens (1.8 m high × 1.5 m long × 1.5 m wide each) in which they were allotted 4 dietary treatments (0, 2.5, 5, and 10% MSM), each with 8 replicate pens of 10 birds, for 28 days. The pen was the experimental unit. Fresh feed and water were ad libitum supplied throughout the duration of the trial. The feeding trial was carried out in a deep-litter system in a broiler house wherein temperature and ventilation were controlled by opening curtains during the day (0800 h to 1700 h). Daily house temperature was maintained in the range of 18 to 21 °C and humidity at 40 to 60%, with a multi-metre device used to monitor them.

### Growth performance

Average feed intake (AFI) was calculated by subtracting the weight of leftover feed from the weight of feed offered and dividing the difference by the total number of birds per pen. Body weight was initially measured on d21 and thereafter weekly by weighing all the birds in each pen using a weighing balance. Body weights were used to calculate average body weight gain (ABWG) according to the equation:

$$ABWG\ \left(t0,T\right)=W\ (T)\hbox{--} W\ (t0)$$

where *t*0 = initial time (days); *T* = final time, *W* (*T*) = final body weight (g), and *W* (*t0*) = initial body weight (g). Weekly feed conversion efficiency (FCE) was calculated as ABWG divided by AFI.

### Carcass characteristics and internal organs

Following 12 h of feed withdrawal, 4 birds were randomly selected from each replicate pen on d50, electrically stunned, bled, and de-feathered. Hot carcass weight (HCW) was measured by weighing eviscerated carcasses within 45 min after slaughter, whilst cold carcass weight (CCW) was measured after chilling the carcasses at 4 °C for 24 h. The weight and length of various carcass cuts (breast, wing, drumstick and thigh) and internal organs (gizzard, proventriculus, spleen, liver, duodenum, jejunum, ileum, caecum and colon) were measured using a digital scale and tape.

### Meat quality

Breast muscle pH and temperature were measured 45 min post-slaughter and 24 h after chilling (4 °C) using a corning Model 4 pH-temperature metre (Corning Glass Works, Medfirld, MA), whilst meat colour (lightness L*, redness a*, and yellowness b*) was measured within 45 min and 24 h after euthanasia using a colorimeter (Minolta, Tokyo, Japan). Shear force was determined using a Meullent-Owens razor shear blade whilst drip loss, water holding capacity, and cooking loss were measured according to Cheng et al. (2019).

### Haemato-biochemistry

On d 50, blood was collected from 2 birds per pen (16 per treatment) from the wing vein using a 21-gauge needle. It was placed into purple-top EDTA-coated vacutainer tubes and analysed for haematology using an automated IDEXX LaserCyte Hematology Analyzer (IDEXX Laboratories (Pty) Ltd., Johannesburg, South Africa), whilst blood collected into red top Vacuette® Serum Clot Activator tubes without EDTA (Greiner Bio-One, GmbH, Frickenhausen, Germany) was analysed for biochemistry using an automated IDEXX Vet Test Chemistry Analyzer (IDEXX Laboratories (Pty) Ltd., Johannesburg, South Africa).

### Statistical analysis

All data were tested for normality using Levene test (Levene, 1960) and homogeneity of variance using Shapiro Wilkinson test (Shapiro and Wilk, 1965). Then, normally distributed data including weekly ADWG, FI, FCE, weights, and lengths of carcass cuts and internal organs, haemato-biochemistry, and meat quality were analysed for linear and quadratic effects by employing polynomial contrasts. The optimum dietary inclusion level of MSM was estimated using response surface regression analysis (SAS, 2002–2012 n.d.) according to the quadratic model: *y* = *ax*^2^ + *bx* + *c*; where *y* = response variable, *a* and *b* = coefficients of the quadratic equation, *c* = intercept, *x* = MSM level (%), and − *b*/2*a* = *x* value for optimal response. Also, weekly ADWG, FI, and FCE data were analysed as repeated measures to determine the diet by week interaction effect, whilst the rest of data (overall ADWG, FI, and FCE; weights and lengths of carcass cuts and internal organs; haemato-biochemistry; and meat quality) were analysed using the general linear models procedure of SAS (2002–2012) in a CRD, with diet being the only main factor. Least square means were compared using the probability of difference option and differences among them deemed significant at *P* ≤ 0.05.

## Results and discussion

Our results showed that dietary MSM did not affect (*P* > 0.05) growth performance indices (Table 2), weights and lengths of carcass cuts and internal organs (Table 3), haemato-biochemistry (Tables 4 and 5), and meat quality (Tables 6 and 7) measurements. The only exception was MSM-induced linear increase in the weight of the duodenum (*y* = 0.08 (± 0.661)*x* + 15.78 (± 1.302); *R*^2^ = 0.141; *P* < 0.05) (Table 3) and quadratic increase in serum phosphorus (*y* = 0.01 (0.006)*x*^2^ − 0.10 (0.065)*x* + 2.79 (0.126); *R*^2^ = 0.153; *P* = 0.05) (Table 5) in contrast to a quadratic decrease in procalcitonin (*y* = 0.01 (± 0.003)*x*^2^ − 0.08 (± 0.029)*x* + 0.49 (± 0.054); *R*^2^ = 0.2622; *P* < 0.01) (Table 4), linear and quadratic decrease in serum total protein [*y* = − 2.17 (± 0.639)*x* + 41.54 (± 1.252), *R*^2^ = 0.131, *P* < 0.05; and *y* = 0.18 (± 0.061)*x*^2^ − 2.17 (± 0.639)*x* + 41.54 (± 1.252), *R*^2^ = 0.237, *P* < 0.01], quadratic decrease in serum albumin (*y* = 0.09 (± 0.043)*x*^2^ − 0.74 (± 0.456)*x* + 14.67 (± 0.893); *R*^2^ = 0.1654; *P* < 0.05), and a quadratic decrease in serum bilirubin (*y* = 0.14 (± 0.035)*x*^2^ − 1.47 (± 0.364)*x* + 7.11 (± 0.712); *R*^2^ = 0.4107; *P* = 0.001) (Table 5).

**Table 2:** The effect of dietary MSM inclusion on growth performance parameters of Cobb 500 broilers

		Dietary MSM inclusion level					*P* value	
		0%	2.5%	5%	10%	SEM	Linear	Quadratic
IBW (g)		885	879	904	916	183.0	0.145	0.851
ADWG								
	Week 4	78.6	69.0	69.4	71.9	6.32	0.524	0.356
	Week 5	54.2	65.4	68.5	73.9	10.38	0.239	0.611
	Week 6	79.9	96.8	70.8	116	33.51	0.950	0.516
ADFI								
	Week 4	125.9	130.7	135.6	133.6	4.81	0.224	0.373
	Week 5	168.6	160.6	173.3	178.8	8.81	0.219	0.618
	Week 6	184.4	195.4	179	205.8	17.40	0.495	0.633
FCE								
	Week 4	0.62	0.53	0.52	0.54	0.04	0.272	0.223
	Week 5	0.32	0.41	0.39	0.43	0.06	0.346	0.599
	Week 6	0.50	0.50	0.60	0.60	0.28	0.760	0.259

*ADWG*, average daily body weight gain; *ADFI*, average daily feed intake; *FCE*, feed conversion efficiency; *MSM*, mucuna seed meal; *SEM*, standard error of the mean based on a pooled estimate of variation (*n* = 80)

**Table 3:** The effect of dietary MSM inclusion on weights and lengths of carcass cuts and internal organs of Cobb 500 broilers

	Dietary MSM inclusion level					*P* value	
Parameters	0%	2.5%	5%	10%	SEM	Linear	Quadratic
HCW (g)	2231	2198	3056	2705	513.72	0.399	0.524
CCW (g)	2140	2052	2956	2111	394.39	0.864	0.213
Wing length (cm)	21.1	21.0	21.6	20.9	0.26	0.829	0.154
Wing weight (g)	113	113	98.8	118	7.73	0.727	0.174
Drumstick length (cm)	12.0	12.0	12.2	12.0	0.18	0.913	0.322
Drumstick weight (g)	132	133	116	133	9.22	0.939	0.302
Thigh length (cm)	8.70	9.20	8.60	8.90	0.39	0.959	0.954
Thigh weight (g)	169	152	149	141	12.11	0.132	0.525
Breast length (cm)	24.5	18.3	18.7	24.6	4.15	0.779	0.165
Breast weight (g)	401	409	407	409	12.72	0.749	0.805
Spleen (g)	3.00	3.19	3.04	2.85	0.22	0.466	0.527
Liver (g)	48.5	46.7	53.1	47.6	1.88	0.915	0.178
Gizzard (g)	38.9	38.3	40.5	39.8	1.47	0.511	0.737
Proventriculus (g)	9.20	8.59	9.62	9.86	0.41	0.864	0.210
Duodenum length (cm)	71.9	66.5	78.4	76.2	3.73	0.191	0.851
Duodenum weight (g)	15.6^b^	18.1^ab^	18.3^ab^	20.0^a^	1.37	0.036	0.578
Jejunum length (cm)	68.44	68.1	71.3	75.2	4.12	0.186	0.799
Jejunum weight (g)	28.1	28.6	29.3	30.5	1.23	0.133	0.997
Ileum length (cm)	20.9	21.9	22.3	22.2	1.62	0.599	0.673
Ileum weight (g)	22.0	31.1	25.0	23.9	3.41	0.868	0.251
Caecum length (cm)	4.55	4.62	5.50	5.36	0.45	0.141	0.503
Caecum weight (g)	13.2	20.4	12.3	12.4	3.67	0.505	0.578
Colon length (cm)	4.60	4.60	5.50	5.40	0.20	0.667	0.428
Colon weight (g)	1.60	2.20	1.80	1.90	0.26	0.787	0.408

*CCW*, cold carcass weight; *HCW*, hot carcass weight; *MSM*, mucuna seed meal; *SEM*, standard error of the mean based on a pooled estimate of variation (*n* = 80)

**Table 4:** The effect of dietary MSM inclusion on haematology of Cobb 500 broilers

	Dietary MSM inclusion level					*P* value	
Parameters	0%	2.5%	5%	10%	SEM	Linear	Quadratic
Red blood cells (× 10^12^/L)	2.60	2.30	2.40	2.10	0.31	0.309	0.899
Haematocrit (L/L)	14.5	14.4	13.1	13.4	1.71	0.606	0.779
Haemoglobin (g/dL)	9.30	9.80	9.60	9.50	0.44	0.841	0.390
MCV (fL)	57.9	56.4	56.5	59.4	2.60	0.426	0.094
MCH (pg)	43.4	47.8	51.6	49.0	5.98	0.503	0.679
RCDW (%)	28.6	28.0	28.4	27.1	1.04	0.322	0.689
Reticulocytes (%)	129	218	155	177	36.1	0.472	0.461
WBC (× 10^9^/L)	67.3	129	137	117	2.72	0.289	0.250
Heterophils (× 10^9^/L)	3.70	4.00	4.40	4.70	0.87	0.266	0.487
Lymphocytes (× 10^9^/L)	72.42	112	131	103	0.29	0.518	0.467
Monocytes (× 10^9^/L)	4.90	4.60	4.30	4.50	1.17	0.907	0.951
Eosinophils (× 10^9^/L)	6.89	10.6	11.2	10.2	0.15	0.169	0.138
Basophils (×10^9^/L)	1.28	1.23	1.31	1.75	0.28	0.264	0.356
Platelet (K/μL)	1755	1740	1598	2195	308.29	0.296	0.1450
MPV (fL)	3.20	3.89	4.16	2.42	0.79	0.413	0.064
PDW (%)	15.0	16.8	17.9	15.1	1.33	0.907	0.334
Procalcitonin (%)	0.49^a^	0.40^a^	0.37^b^	0.59^a^	0.06	0.142	0.005

*MCH*, mean corpuscular haemoglobin; *MCV*, mean corpuscular volume; *MSM*, mucuna seed meal; *MPV*, mean platelet volume; *PDW*, platelet distribution width; *RCDW*, red cell distribution width; *WBC*, white blood cells; *SEM*, standard error of the mean based on a pooled estimate of variation (*n* = 80)

**Table 5:** The effect of dietary MSM inclusion on serum biochemistry of Cobb 500 broilers

Parameters	Dietary MSM inclusion level					*P* value	
	0%	2.5%	5%	10%	SEM	Linear	Quadratic
Symmetric dimethylarginine (μg/dL)	31.7	43.6	34.7	34.4	1.30	0.764	0.155
Blood urea nitrogen (mmol/L)	0.62	0.60	0.60	0.61	0.01	0.999	0.083
Phosphorus (mmol/L)	2.70	2.77	2.45	3.00	0.04	0.174	0.050
Calcium (mmol/L)	6.51	2.42	2.39	2.39	0.04	0.259	0.317
Total protein (g/L)	41.5^a^	38.0^b^	35.8^b^	37.6^b^	0.50	0.044	0.009
Albumin (g/L)	14.7^a^	13.8^a^	13.4^b^	15.9^a^	0.40	0.133	0.039
Globulin (g/L)	26.5	25.3	22.8	23.7	0.50	0.083	0.378
Alkaline phosphatase (U/L)	232	234	242	259	8.60	0.265	0.469
Gamma-glutamyl transferase (U/L)	12.8	4.70	6.79	31.6	8.55	0.142	0.120
Bilirubin (μmol/L)	6.80^a^	5.30^a^	2.60^b^	6.40^a^	0.30	0.427	0.001

*MSM*, mucuna seed meal; *SEM*, standard error of the mean based on a pooled estimate of variation (*n* = 80)

**Table 6:** The effect of dietary MSM inclusion on meat quality parameters of Cobb 500 broilers

	Dietary MSM inclusion level					*P* value	
Parameters	0%	2.5%	5%	10%	SEM	Linear	Quadratic
*45 min*							
pH	6.00	6.00	6.00	6.00	0.7	0.673	0.970
Temperature (°C)	27.7	25.6	27.4	27.7	0.78	0.532	0.318
L*	63.2	63.5	63.3	63.1	1.14	0.863	0.862
a*	2.80	2.70	3.10	3.10	0.38	0.454	0.868
b*	12.4	11.2	12.4	12.1	0.63	0.952	0.728
*24 h*							
pH	5.90	5.90	8.90	5.90	0.02	0.367	0.662
Temperature (°C)	14.4	14.5	14.5	14.3	0.37	0.757	0.729
L*	53.0	53.5	53.7	51.0	1.78	0.384	0.427
a*	2.10	2.50	1.90	1.70	0.30	0.207	0.685
b*	10.9	9.80	9.80	9.50	0.56	0.143	0.387

24 h refers to meat quality parameters 24 h after slaughter (i.e., on the cold carcass); 45 min refers to meat quality parameters 45 min after slaughter; *MSM*, mucuna seed meal; *SEM*, standard error of the mean based on a pooled estimate of variation (*n* = 80)

**Table 7:** The effect of dietary MSM inclusion on meat quality parameters of Cobb 500 broilers

	Dietary MSM inclusion level					*P* value	
Parameter	0%	2.5%	5%	10%	SEM	Linear	Quadratic
Drip loss	0.40	0.40	0.50	0.70	0.19	0.364	0.864
Cooking loss	30.0	31.2	31.6	29.9	0.70	0.750	0.055
Shear force (N)	5.40	5.90	5.70	6.20	0.35	0.157	0.931
WHC	84.1	85.2	79.2	84.1	3.16	0.984	0.340

*MSM*, mucuna seed meal; *WHC*, water holding capacity; *SEM*, standard error of the mean based on a pooled estimate of variation (*n* = 80)

These results suggest that MSM could be supplemented up to 10% in broiler finisher diets without compromising bird performance, carcass cuts and internal organs, haematology, and meat quality. The results are not unexpected considering the low dietary inclusion levels of MSM employed in the current study. In contrast, the literature mostly reports deleterious effects of dietary MSM on broiler growth performance, carcass characteristics, internal organs, and meat quality when the legume seed meal is included at high levels (≥ 10 to 30%) as replacement for SBM (Carmen et al., 1999; Jayaweera et al., 2007; Mthana et al., 2022). Even a dietary level as low as 5% has previously elicited detrimental effects in finisher broilers (Osei and Dei, 1998). It is only when processed that high (16 to 25%) MSM inclusion levels induced no untoward effects in broilers (Vadivel and Pugalenthi, 2010b; Sarmiento-Franco et al., 2019).

Interestingly, our results also showed increased serum phosphorus in broilers fed MSM-containing diets. This corroborates a report of mucuna seeds having the highest content of phosphorus (244.6 mg/100g) of all other minerals (Bhat et al., 2007) with a calcium to phosphorus ratio of 2.28, indicating that the seeds would be a suitable source of the macro-mineral for bone development in broilers. Notwithstanding, a previous study showed decreased blood phosphorus and alkaline phosphatase (hypophosphataemia) in rats following consumption of unprocessed MSM-containing (10%) diets (Huisden et al., 2019). The reason for the differences in animal responses to dietary MSM between the current study and that of Huisden et al. (2019) is unknown. Hence, it would be of interest to further investigate blood (serum) phosphorus and other minerals in broilers fed MSM-supplemented diets in future.

Notwithstanding, our data also demonstrated deleterious effects of dietary MSM on entero-physio-metabolic processes particularly when included at 5 and 10% levels, with most of these effects elicited in blood serum components. In this regard, the observed increase in the weight of the duodenum with incremental dietary levels of MSM (Table 3) points to the presence of toxic phytochemicals most likely L-DOPA in the legume seed meal, if not the high dietary fibre content (Agyekum et al., 2012) (Table 1). Osei and Dei (1998) observed similar hypertrophic effects on the intestine in broilers fed high (15%) MSM-containing diets. Also, other studies observed increased lengths of the duodenum and other intestinal segments due to consumption of diets supplemented with MSM (Oloruntola et al., 2018) and tannin-rich leguminous leaf meals (Miya et al., 2020), in addition to dietary fibre stimulation (Wu et al., 2004) in broilers. However, it is interesting that the relative change in duodenal weight did not influence carcass weights, implying that the observed enteric weight change was merely an adaptation mechanism.

The observation of quadratic decreases in procalcitonin and serum total protein, albumin, and bilirubin appears to be congruent with the observed increase in the weight of the duodenum and suggests MSM-induced enteric and liver toxicity particularly at 10% inclusion level. Produced by liver neuroendocrine cells and macrophages (Matzaraki et al., 2007), increased blood procalcitonin is indicative of liver injury (Rule et al., 2015), and this appears to have been more intense at 10% MSM inclusion level. Also, serum total protein (composed mainly of albumin, globulins, and other proteins) is ordinarily measured as a biomarker of the body’s nutritional status and liver function, with its levels usually decreased in liver dysfunction-associated conditions such as ageing and malnutrition (Furruqh et al., 2004; Sabatino et al., 2017). Constituting 65% of serum total proteins, albumin is a biomarker for malnutrition and is responsible for the transportation, among other substances, of unconjugated bilirubin (Tian et al., 2014). Decreased serum bilirubin levels, on the other hand, occur in physiological conditions associated with oxidative stress and inflammation (Salomone et al., 2013; Tian et al., 2018). It would therefore appear that dietary consumption of high (10%) L-DOPA-infested MSM induced hepatotoxicity that led to protein undernutrition alongside compromised production of albumin, hence poor transportation and decreased serum levels, of bilirubin in broilers. Indeed, there is a positive correlation between procalcitonin and total bilirubin level (Qu et al., 2016) as well as serum total protein and bilirubin (Jonathan et al., 2021). Otherwise, the observed concentrations of blood procalcitonin are similar to those (0.40 to 0.49%) of Matshogo et al. (2021) whilst serum total protein values are within normal ranges (25.0 to 45.0 g/L) for broiler chickens (Harr et al. 2002) and serum bilirubin values are slightly lower but comparable with those in the literature (3.2 to 5.0 μmol/L) (Egbu et al., 2022).

## Conclusion

Our results showed that dietary MSM feeding did not affect growth performance, carcass characteristics, internal organs, as well as haemato-biochemical and meat quality characteristics. Dietary MSM increased duodenum weight and serum phosphorus and decreased procalcitonin and serum levels of total protein, albumin, and bilirubin. The MSM could therefore be supplemented up to 10% without compromising growth performance, carcass traits, internal organs, haemato-biochemistry, and meat quality in finisher broiler diets.

## Data Availability

The datasets generated during the current study are not publicly available due to cooperating producer privacy and confidentiality but are available on request from the corresponding author.
